# Intrafibrillar mineralization of type I collagen by micelle-loaded amorphous calcium phosphate nanoparticles†

**DOI:** 10.1039/d3ra01321a

**Published:** 2023-04-14

**Authors:** Hongyu Xie, Jian Sun, Fangfang Xie, Shengbin He

**Affiliations:** a College of Stomatology, Hospital of Stomatology, Guangxi Medical University Nanning Guangxi 530021 P. R. China xiedualfang@163.com; b Key Laboratory of Longevity and Aging-related Diseases of Chinese Ministry of Education, Guangxi Colleges and Universities Key Laboratory of Biological Molecular Medicine Research, School of Basic Medical Sciences, Guangxi Medical University Nanning Guangxi 530021 P. R. China comhsb@163.com

## Abstract

Mineralization of type I collagen fibrils is highly desired for artificial bone preparation and teeth repairing. Generally, amorphous calcium phosphate (ACP) combined with non-collagenous protein analogue (NCPA) were used for biomimetic remineralization of collagen fibrils. However, the ACP was likely to aggregate to form larger particles that could not infiltrate into the gaps of the collagen for intrafibrillar mineralization, and the poor storage stability of ACP has challenged its practical applications. To address this question, here we assembled ACP that was stabilized by carboxylated polyamidoamine (CPAMAM) at a pH of 6.5 to form dispersed nanoparticles of 25 nm in size, which was named as ACP/CPAMAM. The ACP/CPAMAM nanoparticles were further loaded into micelles composed of polysorbate and polyethylene glycol (PEG) to further improve their storage stability. The micelle-loaded ACP/CPAMAM particles could maintain their amorphous phase after storage for 12 months. During the mineralization of collagen fibrils, isopropanol (IPA) was introduced to dissolve the micelles and release the ACP/CPAMAM nanoparticles. By using micelle-loaded ACP/CPAMAM, good intrafibrillar mineralization of type I collagen was demonstrated. This work provides novel methods for preparing ACP nanoparticles with good storage stability and controllable release for intrafibrillar mineralization.

## Abstract 2

Intrafibrillar mineralization of collagen by micelle-loaded amorphous calcium phosphate.

## Graphical abstract

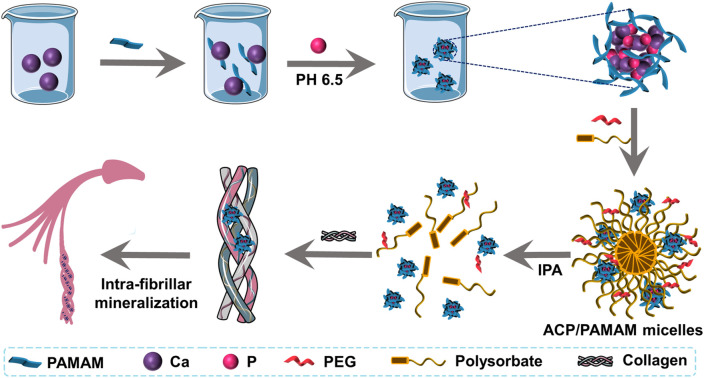

## 1. Introduction

Biominerals such as teeth and bones are composites of an inorganic component, predominantly hydroxyapatite (HAP), and an organic component, predominantly type I collagen. The HAP nanocrystalline fits within the gap zones of type I collagen molecules, endowing the biominerals with reasonable hardness and toughness to perform their skeleton functions. Demineralization happened frequently in dental caries, dental erosion, and bone lesions.^1–4^ Once the biominerals begin to be demineralized pathologically or intentionally, collagen fibrils become exposed to endogenous protease and are degraded. As a result, the biomineral structure would be disintegrated, and its hardness and toughness were decreased. To solve this problem, remineralization of collagen has been widely investigated as thoroughly methods for teeth repairing and bone reconstruction.^5–8^

The most widely used methods for remineralization of collagen were to develop calcium phosphate (CaP) microparticles of 1–5 μm in size or amorphous calcium phosphate (ACP) nanoparticles of 30–500 nm in size.^9–11^ Both of them could release Ca and P ions to the peripheral matrix of collagen for the subsequent mineralization. The ACP nanoparticles had higher surface area to volume ratio and released higher levels of Ca and P ions than CaP microparticles, and hence became very popular in recent years. Because ACP would transform easily into a thermodynamically more stable HAP crystalloid, biomimetic analogs of noncollagenous proteins were generally used to transitorily stabilize the ACP and guide the nucleation during crystallization process.^12–15^ However, the ACP was likely to aggregate to form larger particles that could not infiltrate directly into the gaps of the collagen (40 nm) for intrafibrillar mineralization, and the released Ca and P ions deposited outside the fibrillar collagen to form HAP for extrafibrillar mineralization.^16,17^ Additionally, the poor storage stability of ACP has challenged its practical applications.

**Fig. 1:**
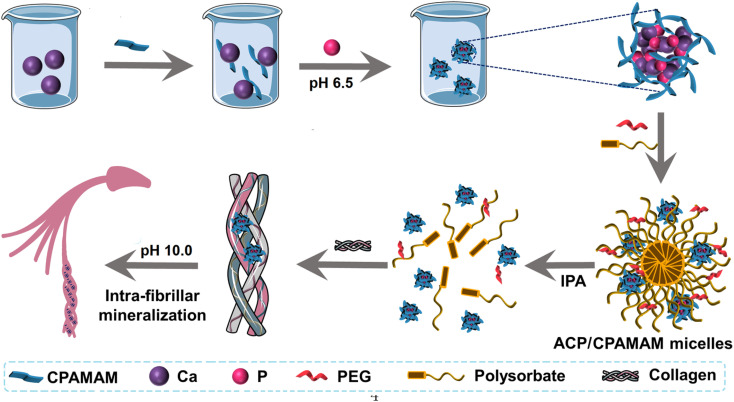
Schematic diagrams for assembling micelle-loaded ACP/CPAMAM nanoparticles.

Intrafibrillar mineralization of collagen not only increases the mechanical stability of the biomineral, but also protects the collagen molecules from external challenges, such as bacterial degradation and endogenous enzymolysis.^18–21^ In the present work, we assembled ACP of about 25 nm in size, which were stabilized by carboxylated polyamidoamine (CPAMAM) to form dispersed ACP/CPAMAM nanoparticles, as shown in Fig. 1. The ACP/CPAMAM nanoparticles were further loaded into micelles composed of polysorbate and polyethylene glycol (PEG) to further improve their storage stability. During the mineralization of collagen fibrils, isopropyl alcohol (IPA) was introduced to dissolve the micelles and release the ACP/CPAMAM nanoparticles. The small sizes of the ACP/CPAMAM nanoparticles enable them to infiltrate directly into the gaps of the collagen for intrafibrillar mineralization. By using the micelle-loaded ACP/CPAMAM nanoparticles, good intrafibrillar mineralization of type I collagen was demonstrated.

## 2. Materials and methods

### 2.1. Synthesis of ACP/CPAMAM nanoparticles

CPAMAM of 158 mg was added to 25 mL CaCl_2_ solution (10 mM). The mixture was incubated at room temperature for 24 h, and its pH was adjusted to 6.0 by NaOH/HCl. Then 25 mL Na_2_HPO_4_ (6 mM) was added to the mixture. The mixture was stirred for various reaction times (1 min, 30 min, 60 min, 120 min) at room temperature. The final mineralization solution exhibited a pH of 6.5 without any precipitate. The mixed solutions were then subjected to ultracentrifugation (Beckman Coulter, Fullerton, CA, USA) at 100 000 g for 1 h at 4 °C, and the supernatant was removed.

The surface characteristics and corresponding histograms representing diameter size distribution of the ACP/CPAMAM nanoparticles were observed using TEM (Talos F200X, FEI, USA). Prior to the observations, the samples were prepared by placing a drop of ACP/CPAMAM solution on a 400 formvar-coated Ni transmission electron microscope grid and allowing it to air-dry at 25 °C. Meanwhile, the distribution of calcium and phosphorus inside the samples was determined *via* STEM-EDX mapping (Talos F200X, FEI, USA), and the crystallinity of the minerals was confirmed using SAED (Talos F200X, FEI, USA). DLS measurements were used to obtain zeta potentials (Nano ZS90, Malvern, the UK) of the samples. Additionally, the chemical bonds of the ACP/CPAMAM nanoparticles were analyzed using Fourier transform infrared (FTIR) spectroscopy (Nicolet iS10, Thermo Fisher Scientific, USA).

### 2.2. Preparation of micelles-loaded ACP/CPAMAM nanoparticles

PEG-6000 (30 mg mL^−1^, Shenggong Bioengineering Co., Ltd., China) was dissolved in the ACP/CPAMAM solution and reacted for 30 min. Polysorbate (ranging from 50, 100, 350, 500, and 1000 mg mL^−1^) was slowly added to the solution, and the mixture was stirred for 3 h at room temperature. The micelles were then obtained by centrifuging the micelles solution at 100 000 rcf for 1 h at 4 °C to remove the supernatant containing the free calcium and phosphate ions. After centrifugation, the supernatant was collected to determine the concentration of calcium ions using inductively coupled plasma-optical emission spectrometry (ICP-OES) (73, Agilent, USA) to calculate the encapsulation efficiency (EE) through the following formula:1
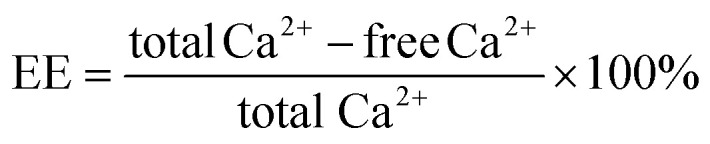


The morphology of micelle-loaded ACP/CPAMAM and micelle-loaded ACP was observed using TEM; the distribution of ACP/CPAMAM nanoparticles in the samples was determined *via* STEM-EDX mapping. The crystallinity of the minerals was validated using SAED. The hydrodynamic diameter and the zeta potentials of the samples were measured using a Zeta sizer Nano ZS detector. Additionally, the chemical bonds of the micelle-loaded ACP/CPAMAM and empty micelles were analyzed using a Raman DXR spectrometer (Thermo Scientific, Waltham, MA, USA).

### 2.3. Release of ACP/CPAMAM nanoparticles mediated *via* IPA

To destroy the structure of micelles and release the ACP/CPAMAM nanoparticles as the mineralization medium, we added IPA of various concentrations (0.1, 0.2, 0.4, and 0.6 g mL^−1^) into the micelle-loaded ACP/CPAMAM at pH 6.5, the solutions were then subjected to vortex mixing for 10 min, followed by ultracentrifugation at 10 000 rcf for 60 min at 4 °C. The final concentrations of calcium ions in the solution were then measured *via* ICP-OES, and the state of the ACP/CPAMAM nanoparticles was observed *via* TEM. Additionally, DLS measurements were used to obtain zeta potentials of the samples.

### 2.4. Preparation and intrafibrillar mineralization of type I collagen by micelle-loaded ACP/CPAMAM nanoparticles

Collagen fibrils were self-assembled by dissolving type I collagen in 0.02 M hydrochloric acid overnight at 4 °C, and 2 μg mL^−1^ phenol red (Shenggong Bioengineering Co., Ltd., China) was added to the solution. Subsequently, 65 μL collagen solution was placed on a Petri dish, on which a 400-mesh, carbon-coated, formvar-coated Ni transmission electron microscope grid (Zhongjingkeyi, P. R. China) was placed. The collagen solution was then neutralized using 25% ammonium hydroxide solution (v/v), which was indicated by the color change from red to yellow of the pH indicator, and the neutralized collagen solution was left to gel *via* incubating at 37 °C for 24 h. The as-constructed collagen was then crosslinked using a 0.03 M 1-ethyl-3-(3-dimethylaminopropyl)-carbodiimide (EDC)/0.06 M *N*-hydroxysuccinimide (Yuanye, Bioengineering, Co., Ltd., China) solution for 4 h. For the subsequent tests, the grids were briefly cleaned three times using deionized water and air-dried. The structures and diameters of collagen fibrils were then observed and measured using TEM.

The collagen-coated grids were floated upside down over 200 μL of the ACP nanoparticles and ACP/CPAMAM nanoparticles mediated *via* IPA in a chamber with 100% humidity for 3 d, respectively, and the mineralization mediums was replaced every 24 h. Subsequently, the mineralization of collagen fibrils was observed using TEM at 80 kV for 72 h, after which the grids were washed three times with deionized water and then dried in air. The remineralization of collagen fibrils and the crystal properties of the formed minerals inside of the collagen fibrils were subsequently observed and analyzed using TEM and STEM-EDX mapping.

### 2.5. Extrafibrillar mineralization of type I collagen fibrils tablet by micelle-loaded ACP/CPAMAM nanoparticles

Twenty-four sound human third molars were obtained and approved by the Ethics Committee of the Department of Oral and Maxillofacial Surgery of the Affiliated Stomatological Hospital of Guangxi Medical University with a scalpel blade removed the organic contaminants. Then the teeth were treated with 3% sodium hypochlorite to remove bacteria, which were stored in 0.5% thymol at 4 °C and used in a month. Using a low-speed water cooled diamond saw (Isomet, Buehler, Lake Bluff, IL, USA), the teeth were cut perpendicular to the long axis of tooth, above the cement–enamel junction (CEJ) to prepared into a 5 × 5 × 1 mm dentin specimen. After polishing and ultrasonic cleaning, the human dentin grinding tablets were prepared, then the samples (*n* = 8) were immersed in 0.5 M EDTA (pH 8.0) (Shenggong Bioengineering Co., Ltd., China) solution at 25 °C for 30 min to remove the smear layer, others were demineralized with 0.5 M EDTA (pH 8.0) (Shenggong Bioengineering Co., Ltd., China) solution at 25 °C for 7d, Further, the samples were immersed in guanidine chloride (GuCl, 4 M) (Shenggong Bioengineering Co., Ltd., China) to remove the non-collagen protein for 24 h. Finally, the specimens (*n* = 8) were rinsed with 1 mL of ACP/CPAMAM nanoparticles mediated *via* IPA in a chamber with 100% humidity with the remineralizing solution was changed every day. After remineralization for 15 days, the dentin discs were washed three times with deionized water and ultrasonicated for 10 min. The qualitatively characterize the demineralization degree of these dentin discs were analyzed using scanning electron microscopy (SEM; SU8020, Hitachi, Japan) operating at 20 kV.

## 3. Results and discussion

### 3.1. ACP/CPAMAM nanoparticles with good monodispersity were synthesized

Intrafibrillar mineralization cannot be achieved by simply immersing collagen fibrils in Ca/P solutions; it requires the use of nucleation inhibitors to stabilize the Ca/P relevant complexes and prevent them from crystallizing outside the fibrils. In biomineralization, non-collagenous protein analogues (NCPAs), such as osteopontin and dentin matrix proteins provide as nucleation inhibitors and/or templates to guide intrafibrillar mineralization orderly.^2,22^ However, they are not economically viable for clinical research due to technical limitations in the extraction and preservation.^23,24^ Carboxylated polyamidoamine (CPAMAM) is a highly-branched polymer characterized by the presence of multiple carboxyls to simulate the NCPA functions. Although the combination of ACP and CPAMAM has been used previously,^10,25,26^ these studies mainly focused on preparing ACP/CPAMAM in alkaline condition (pH 9–10) to form large and aggregated ACP particles. The large and aggregated ACP particles are unfavorable for intrafibrilar mineralization, because they cannot infiltrate into the gaps of the collagen.

**Fig. 2:**
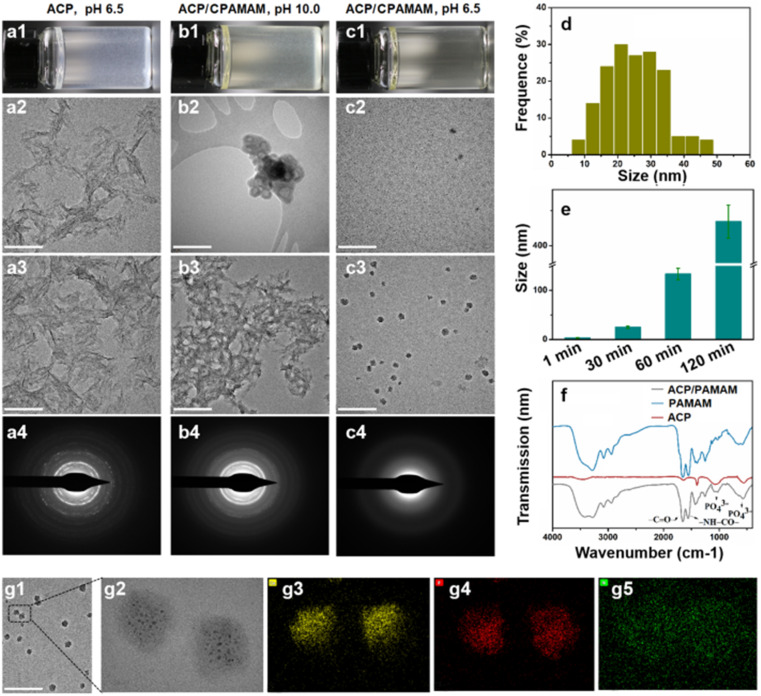
Preparation of ACP nanoparticles with three different formulas. (a1–c1) Reaction mixtures for preparing ACP under weakly acidic condition (a1), ACP/CPAMAM under alkaline condition (b1), and ACP/CPAMAM under weak acidic condition (c1). (a2–c2) Representative TEM images of the above three ACP formulas with reaction time of 1 min. Scale bar: 200 nm. (a3–c3) Representative TEM images of the above three ACP formulas with reaction time of 30 min. (a4–c4) Selected area electron diffraction of the three ACP formulas from a3–c3. (d)Size distribution of ACP/CPAMAM prepared under weak acidic condition with reaction time of 30 min. (e) The average size of the ACP/CPAMAM prepared under weak acidic condition for various reaction times. (f) Infrared spectra of the ACP/CPAMAM. (g1–g5) STEM-EDX mapping for calcium (g3), phosphorus (g4), and nitrogen (g5) of the ACP/CPAMAM nanoparticles.

To obtain ACP nanoparticles with size smaller than the gap of the collagen, here three methods were used for preparing ACP: (1) without stabilizer; (2) with CPAMAM as a stabilizer in alkaline condition (pH = 10.0); (3) with CPAMAM as a stabilizer in weak acidic condition (pH = 6.5). As shown in Fig. 2a1–a4, after mixing Ca and P for 1 min, the Ca/P mixture transformed fleetly to crystal phase, which is similar with the results described previously.^27–29^ Small crystal sediments in the glass container were visible to the eyes (Fig. 2a). When the CPAMAM was used as a stabilizer in alkaline condition, the Ca/P mixture formed ACP nanoparticles of about 30 nm in size, which aggregate to form agglomerates, as shown in Fig. 2b1 and b2. These agglomerates further transformed to crystal phase after incubation for 30 min (Fig. 2b3 and b4). When the CPAMAM was used in weak acidic condition, the ACP formed dispersed nanoparticles of 4 nm in size and no sediments were visible in the glass container, as shown in Fig. 2c1 and c2. These ACP nanoparticles grew to about 25 nm after incubating the Ca/P/CPAMAM mixture for 30 min (Fig. 2c3 and c4). The size distribution of the ACP nanoparticles ranged from 10 to 40 nm (Fig. 2d), which is smaller than the gap of the collagen.

Fig. 2e and ESI Fig. S1† show the average size of the ACP nanoparticles prepared in various reaction times. The size of the ACP nanoparticles could be controlled by changing the reaction time of the Ca/P/CPAMAM mixture. When the reaction time increased to 60 min, the formed ACP nanoparticles were too large to enter the gap of the collagen. Hence, we chose 30 min as the optimal reaction time for the subsequent experiments. The FTIR spectra of ACP/CPAMAM are shown in Fig. 2f. The absorption peaks at 1246, 1517 and 1650 cm^−1^ corresponded to the stretching vibrations of amide bone of the CPAMAM, and the peak at 1075 cm^−1^ was assigned to PO4^3+^. All these characteristic peaks were present in the spectrum for ACP/CPAMAM group. STEM-EDX mapping showed colocalization of calcium (from ACP), phosphorus (from ACP), and nitrogen (from CPAMAM), as shown in Fig. 2g1–g5. Both FTIR and STEM-EDX demonstrated the incorporation of ACP and CPAMAM to form ACP/CPAMAM nanoparticles. These results indicated the ACP nanoparticles with good monodispersity were successfully prepared by using CPAMAM as a stabilizer in weak acidic condition.

### 3.2. The ACP/CPAMAM nanoparticles were loaded into micelles

Although the ACP could be stabilized transitorily by CPAMAM to form dispersed ACP/CPAMAM nanoparticles, they were likely to aggregate after storage for a period of time due to the low zeta potential of the nanoparticles (−6). In order to improve the storage stability of the ACP/CPAMAM nanoparticles and expand their practical applications, we used polysorbate combined with PEG to separate the ACP/CPAMAM nanoparticle from each other. As a surfactant, polysorbate would self-assemble to form micelles in aqueous phase,^30^ during which the ACP/CPAMAM nanoparticles were supposed to be included in the micelles. Meanwhile, PEG was used to stabilize the micelles in aqueous phase.

**Fig. 3:**
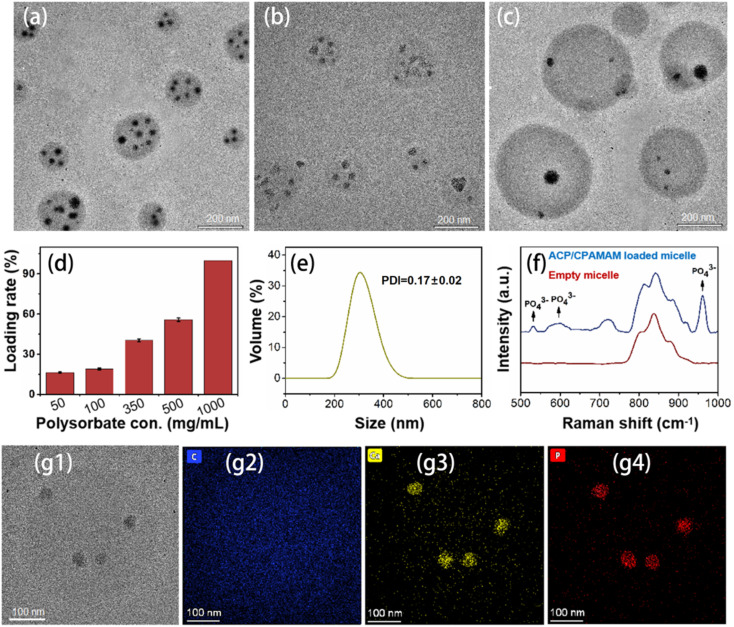
Assembling of micelle-loaded ACP/CPAMAM. (a–c) TEM images of the micelle-loaded ACP/CPAMAM nanoparticles, with polysorbate concentrations of 100 mg mL^−1^ (a), 350 mg mL^−1^ (b), and 500 mg mL^−1^ (c). (d) Loading rate of the ACP/CPAMAM in the micelles under various polysorbate concentrations. (e and f) The size distribution (e) and Raman spectrum (f) of the micelles, with polysorbate concentrations of 350 mg mL^−1^ (g1–g4) STEM-EDX mapping for carbon, calcium and phosphorus of the micelle.

As shown in Fig. 3a, micelles with size ranging from 100 to 250 nm were assembled when polysorbate solution of 100 mg mL^−1^ was ultrasonic agitated with the ACP/CPAMAM for 3 h. The ACP/CPAMAM nanoparticles were successfully loaded into the micelles. The size of the micelles and the loading efficiency of the ACP/CPAMAM in the micelles increased with the increasing of polysorbate concentration, as shown in Fig. 3b–3d. However, when the polysorbate concentration reached to 500 mg mL^−1^, lots of unloaded micelles appeared. Hence, we took 350 mg mL^−1^ as the optimal polysorbate concentration, where a loading rate of 40% and an average size of 303 nm were obtained (Fig. 3e). The Raman peaks for PO_4_^3+^ at 532 cm^−1^, 600 cm^−1^, and 960 cm^−1^ indicated the presence of ACP in the micelles, as shown in Fig. 3f. A representative micelle containing four ACP/CPAMAM nanoparticles was analyzed by STEM-EDX mapping, as shown in Fig. 3g1–g4. The phosphorus and calcium spots in the micelle were colocalized with the ACP/CPAMAM nanoparticles, further indicating the ACP were loaded into the micelles.

### 3.3. The micelles-loaded ACP/CPAMAM nanoparticles showed good storage stability

**Fig. 4:**
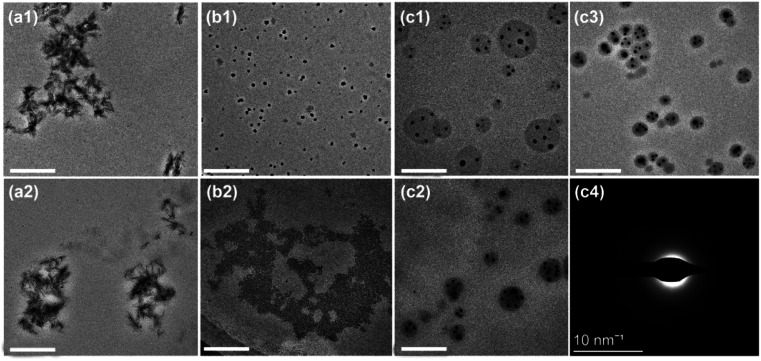
Comparison of storage stability between the ACP/CPAMAM and micelle-loaded ACP/CPAMAM. (a1–c1) Representative TEM images of micelle-loaded ACP (a1), ACP/CPAMAM (b1), and micelle-loaded ACP/CPAMAM (c1) stored for one day. (a2–c2) The above three ACP formulas after storing for 30 days. (c3) Micelle-loaded ACP/CPAMAM after storing for one year. (c4) Selected area electron diffraction of the micelle-loaded ACP/CPAMAM after storing for one year. Scale bar: 200 nm.

The micelles-loaded ACP/CPAMAM nanoparticle solutions were stored at 4 °C in refrigerator for different times. Fig. 4 shows the comparison of storage stability for various ACP formulas. Without CPAMAM, the ACP aggregated and transformed to crystal phase rapidly even in the present of micelle (Fig. 4a1 and a2). The CPAMAM could stabilize the ACP nanoparticles and hence prevented them from crystal phase transition (Fig. 4b1). However, after storage for 30 days, the CPAMAM stabilized ACP (ACP/CPAMAM) nanoparticles aggregated to form sediments (Fig. 4b2), which was no longer acceptable for intrafibrillar mineralization. When the ACP/CPAMAM nanoparticles were loaded into the micelles, the ACP maintained amorphous phase even after storage for 12 months, as shown in Fig. 4c1–c4. These results demonstrated that the micelles could block the collision between the ACP/CPAMAM nanoparticles by separating the particles from each other. Meanwhile, the CPAMAM kept the ACP at amorphous phase. The combined use of CPAMAM and micelle enable us to keep the ACP monodispersed at amorphous phase.

### 3.4. Controlled release of ACP/CPAMAM nanoparticles from the micelles

**Fig. 5:**
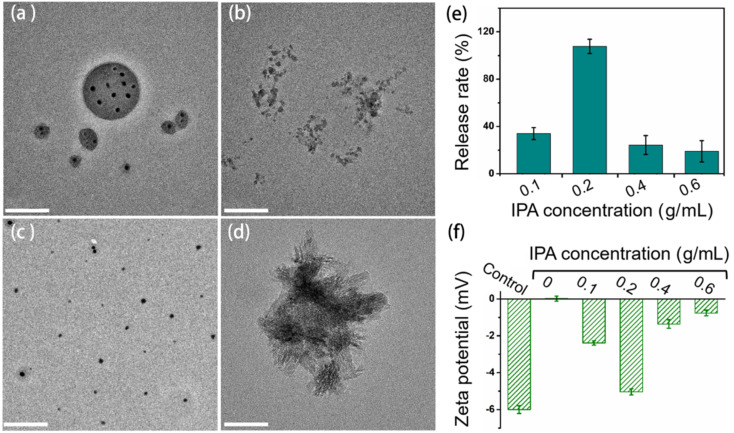
Release of ACP/CPAMAM nanoparticles from the micelles. (a) Micelle-loaded ACP/CPAMAM. (b–d) Micelle-loaded ACP/CPAMAM with the addition of 0.1 g mL^−1^ (b), 0.2 g mL^−1^ (c), and 0.4 g mL^−1^ (d) IPA. Scale bar: 200 nm. (e) Release rate of the ACP/CPAMAM in the micelles under various IPA concentrations. (f) Zeta potentials of the micelle-loaded ACP/CPAMAM solution under various IPA concentrations. Freshly prepared ACP/CPAMAM without micelles was measured as a control.

When the micelle-loaded ACP/CPAMAM is used for mineralization, the ACP/CPAMAM nanoparticles need to be released from the micelles. Isopropanol (IPA), as a lipolytic reagent, could sharply decrease the surface tension of lipoid polysorbate in aqueous phase,^31^ which might lead to the dissolution of the micelles. Hence, we use IPA of various concentrations to destroy the micelles and release the ACP/CPAMAM nanoparticles, as shown in Fig. 5a–5d. When 0.1 g mL^−1^ IPA was added to the micelle-loaded ACP/CPAMAM, some micelles were just under cracking (Fig. 5b). When the IPA increased to 0.2 g mL^−1^, nearly all the ACP nanoparticles released to the solutions (Fig. 5c). The released ACP/CPAMAM particles could be separated from the micelles thorough centrifugation at 10 000 rcf for 60 min, the supernatant of which was subjected to calcium content analysis for release rate determination. Theoretically, there should be a positive correlation between the released ACP/CPAMAM nanoparticles and the IPA concentration used for cracking the micelles. However, it was worth noting that the IPA could accelerate the transformation of ACP from amorphous phase to crystal phase. High IPA concentration led to the rapid agglomeration of the ACP and formation of crystal phase, as shown in Fig. 5d. The agglomeration and crystallization resulted in the low release rate of the ACP (Fig. 5d and e). In addition, the ACP/CPAMAM nanoparticles released by 0.2 g mL^−1^ IPA had a similar zeta potential with that of freshly prepared ACP/CPAMAM nanoparticles, as shown in Fig. 5f, indicating they had similar colloidal stability. These results indicated the release of ACP/CPAMAM nanoparticles from the micelles could be controlled by adding IPA, with an optimal concentration of 0.2 g mL^−1^.

### 3.5. Mineralization of collagen by micelle-loaded ACP/CPAMAM nanoparticles

To demonstrate the mineralization function of the micelle-loaded ACP/CPAMAM, self-assembled type I collagen fibrils were exposed to the micelle-loaded ACP/CPAMAM for 5 days, during which IPA was introduced to release the ACP/CPAMAM nanoparticles from the micelles at pH of 6.5. Then ACP particles smaller than 40 nm initially infiltrate into the gaps of the collagen, as described in Fig. 1. By adjusting the pH of the system from 6.5 to 10.0, the ACP particles transformed into thermodynamically stable HAP.

**Fig. 6:**
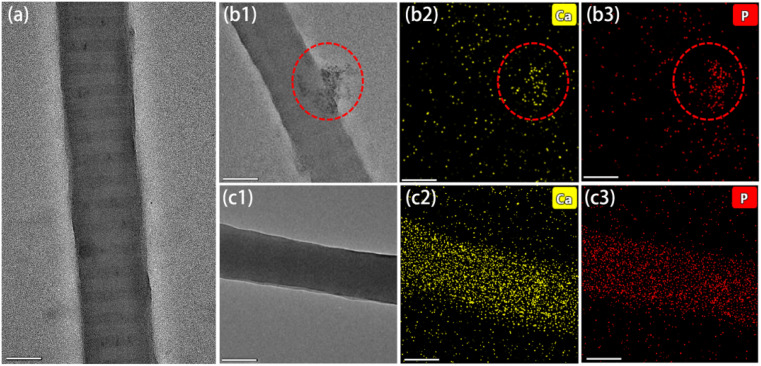
Intrafibrillar mineralization of type I collagen by micelle-loaded ACP/CPAMAM nanoparticles. (a) TEM image of reconstituted collagen fibril without mineralization. (b1–c1) TEM images of reconstituted collagen fibrils mineralized directly by Ca/P mixture (b1, as control), and micelle-loaded ACP/CPAMAM (c1) for 5 days. (b2–c2) and (b3–c3) STEM-EDX mapping for calcium and phosphorus of the above samples. Scale bar: 200 nm.

The reconstituted collagen fibrils without mineralization showed stripe-like gaps, with a size of about 50 nm, as shown in Fig. 6a. When Ca/P mixture (ACP) was directly used for mineralization, the ACP transformed fleetly to crystal phase before entering into the gaps of the collagen, leading to failure in intrafibrillar mineralization. As a result, few calcium and phosphorus dots deposited along with the collagen fibrils, as shown in Fig. 6b1–b3. The red circles in Fig. 6b1–b3 showed the extrafibrillar deposition of HAP crystal, which could not enter the gap of the collagen. When the micelle-loaded ACP/CPAMAM was used, the intrafibrillar mineralization of collagen was accomplished by infiltrating the ACP/CPAMAM nanoparticles into the collagen fibrils *via* gap zones. After intrafibrillar mineralization, Ca and P distributed uniformly through the whole fibril, as shown in Fig. 6c1–c3. As a result, the electron density of the TEM image was uniform and the stripe-like gaps were disappeared (Fig. 6c1).

**Fig. 7:**
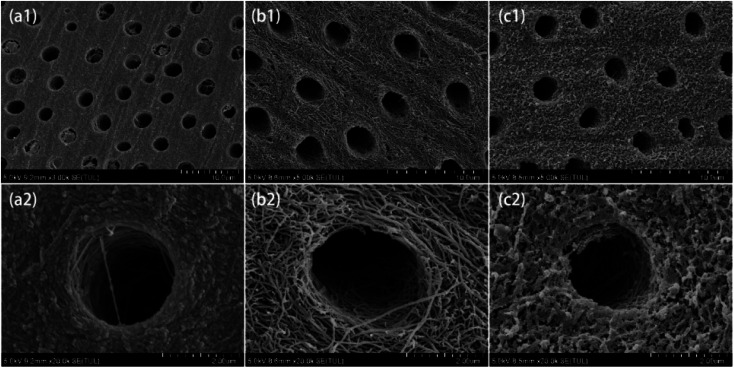
Extrafibrillar mineralization of dentin by micelle-loaded ACP/CPAMAM. (a1, a2) Natural dentin. (b1, b2) Demineralized dentin. (c1, c2) Remineralized dentin, which was mineralized by micelle-loaded ACP/CPAMAM for 15 days. (a2), (b2) and (c2) are the enlarged images of (a1), (b1) and (c1), respectively.

For teeth repairing and bone reconstruction, both intrafibrillar and extrafibrillar mineralizations are expected to obtain artificial biominerals with structure and function as similar to natural ones as possible. Hence, demineralized teeth were used to further evaluate the extrafibrillar mineralization effect of the micelle-loaded ACP/CPAMAM. As shown in Fig. 7a1 and a2, natural dentin showed a dense structure, with type I collagen filled with HAP both internally and externally. Once being demineralized by EDTA, the net-like collagen fibrils were exposed as shown in Fig. 7b1 and b2. After remineralization for 15 days by the micelle-loaded ACP/CPAMAM, the collagen fibril nets were refilled with HAP as shown in Fig. 7c1, c2 and ESI Fig. S2,† indicating the successful extrafibrillar mineralization of the collagen. However, there were still many micropores remaining in the fibril nets, and mineralization time need to be prolonged to cover all the interspaces of the fibrils.

## 4. Conclusions

Mineralization of collagen fibrils is of great importance for assembling biomimetic bones. As the most widely used mineralization reagent, amorphous calcium phosphates (ACPs) were extremely unstable. They are likely to aggregate and transform to crystalline phase before entering into the gaps of intrafibrillar and extrafibrillar collagen. In this study, we firstly assembled ACP nanoparticles, which were stabilized by carboxylated polyamidoamine (CPAMAM) at slight acidic environment to form monodispersed ACP/CPAMAM nanoparticles. The ACP/CPAMAM nanoparticles were further loaded into micelles composed of polysorbate and PEG, which prevent the ACP nanoparticles from aggregation and prolong their storage time. When the micelle-loaded ACP/CPAMAM was used for mineralization of collagen, IPA was added to destroy the micelles and release the ACP/CPAMAM nanoparticles, which then entered the gaps of the collagen for both intrafibrillar and extrafibrillar mineralization. Good mineralization results were demonstrated by *in vivo* extrafibrillar mineralization and *in vitro* intrafibrillar mineralization experiments.

## Author contributions

Hongyu Xie and Jian Sun: investigation & writing. Fangfang Xie: investigation & funding acquisition. Shengbin He: conceptualization, writing-review & editing, project administration.

## Conflicts of interest

No potential conflict of interest is reported by the authors.

## Supplementary Material

RA-013-D3RA01321A-s001
