# Supplementary material for: Intrafibrillar mineralization of type I collagen by micelle-loaded amorphous calcium phosphate nanoparticles
Source: RSC Adv. 2023 Apr 14;13(17):11733–41. doi: 10.1039/d3ra01321a (PMC10103074; doi:10.1039/d3ra01321a)
Supplement: RA-013-D3RA01321A-s001 [file RA-013-D3RA01321A-s001.pdf]

Electronic Supplementary Material (ESI) for RSC Advances.
This journal is © The Royal Society of Chemistry 2023

Supporting Material for

Intrafibrillar mineralization of type I collagen by micelle-loaded amorphous calcium phosphate nanoparticles

Hongyu Xie[a,†], Jian Sun[b,†], Fangfang Xie[a]**, Shengbin He[b]*,

a. College of Stomatology, Hospital of Stomatology, Guangxi Medical University, Nanning, Guangxi 530021, P. R. China.

b. Key Laboratory of Longevity and Aging-related Diseases of Chinese Ministry of Education, Guangxi Colleges and Universities Key Laboratory of Biological Molecular Medicine Research, School of Basic Medical Sciences, Guangxi Medical University, Nanning, Guangxi 530021, P. R. China.

[†]These authors contributed equally to this work.

* Corresponding author.

** Corresponding author.

E-mails addresses: comhsb@163.com (Shengbin He); xiedualfang@163.com (Fangfang Xie)

This Supporting Information inlcudes **Supplementary Figure S1 and Figure S2**.

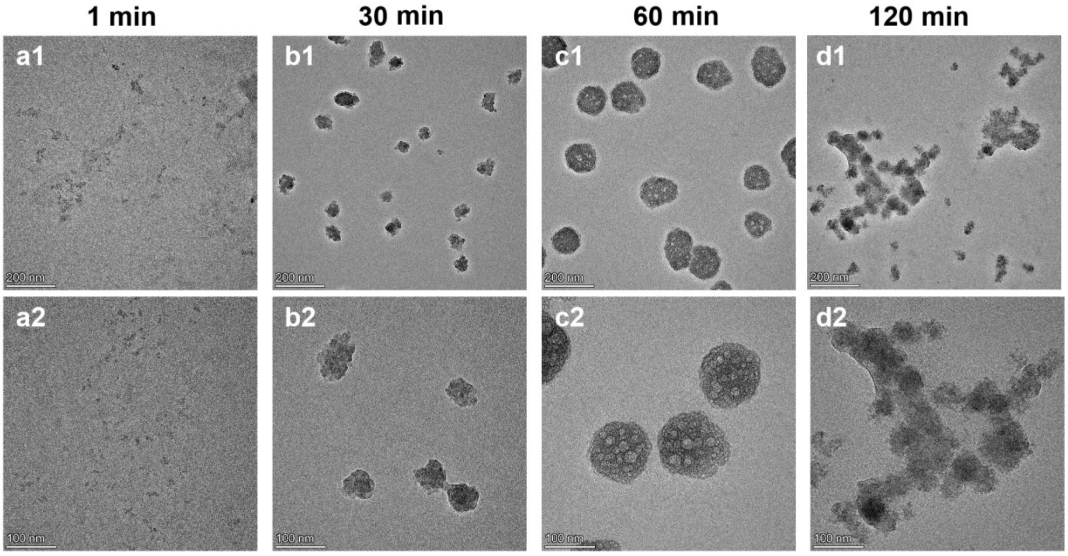

Supplementary Fig. S1 Representative TEM images of the ACP/CPAMAM with various reaction times.

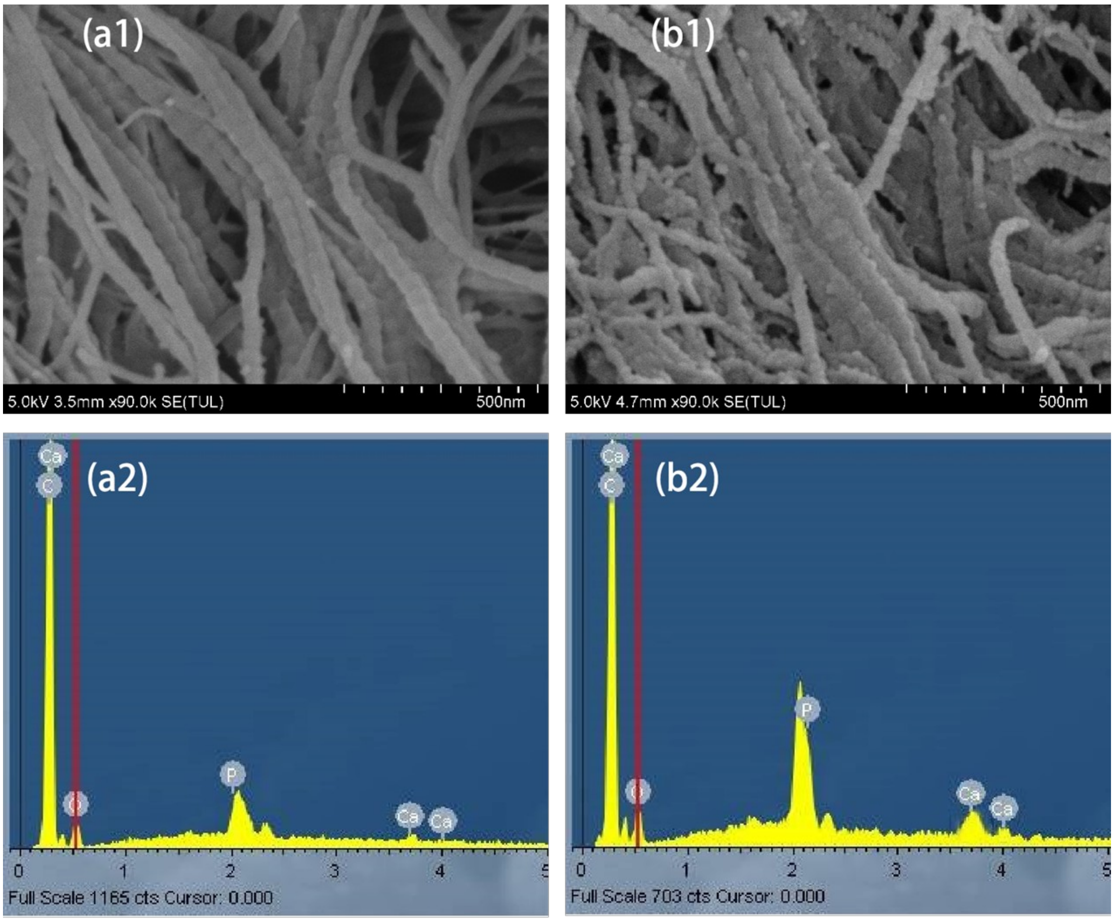

Supplementary Fig. S2. TEM image and energy spectrum analysis of the demineralized dentin (a1-a2) and remineralized dentin (b1-b2).
